# Resin Infiltration for Anterior Teeth Affected by Molar Incisor Hypomineralization in Children and Adolescents: A Clinical Study of Color Masking, Sensitivity, and Aesthetic Perception: A Prospective Single-Arm Interventional Clinical Study

**DOI:** 10.3390/children13010131

**Published:** 2026-01-15

**Authors:** María Dolores Casaña-Ruiz, Mª Ángeles Vello-Ribes, Montserrat Catalá-Pizarro

**Affiliations:** Department of Stomatology, Faculty of Medicine and Dentistry, University of Valencia, 46010 Valencia, Spain; m.angeles.vello@uv.es (M.Á.V.-R.); montserrat.catala@uv.es (M.C.-P.)

**Keywords:** molar incisor hypomineralization, MIH, Resin infiltration, pediatric dentistry, Icon^®^

## Abstract

**Highlights:**

Resin infiltration is a minimally invasive, conservative, and effective approach for managing anterior MIH opacities, producing measurable improvements in color, lesion size, and hypersensitivity. Aesthetic outcomes and patient self-perception improve rapidly after treatment and remain stable over the 6-month follow-up period.

**What are the main findings?**

- Resin infiltration with Icon significantly improves color parameters (with clinically perceptible ΔE changes), reduces lesion size, and decreases hypersensitivity in anterior teeth affected by MIH.
- Patients report a substantial improvement in aesthetic perception following treatment, reaching “good” or “excellent” ratings, although agreement with professional assessments remains low.

**What are the implications of the main findings?**

- Resin infiltration should be considered a first-line, minimally invasive aesthetic treatment option for anterior MIH lesions in children and adolescents, given its clinical efficacy and psychosocial benefits.
- The findings highlight the importance of incorporating patient-reported aesthetic outcomes into clinical evaluation, as patients and professionals may assess treatment success differently, reinforcing a patient-centered clinical approach

**Abstract:**

Background/objective: To evaluate the effectiveness of resin infiltration in managing anterior molar incisor hypomineralization (MIH) defects, focusing on color improvement, lesion size reduction, sensitivity outcomes and patient aesthetic perception. Enamel defects in MIH result from a combination of environmental, systemic, and genetic factors, indicating a multifactorial etiology. These defects, particularly in anterior teeth, pose significant aesthetic and emotional challenges due to their high visibility. This study provides one of the few prospective clinical evaluations of resin infiltration for anterior MIH lesions, assessing not only objective clinical outcomes but also patients’ aesthetic perception. It further introduces a patient-centered approach by comparing aesthetic evaluations made by children and dental professionals over time. Methods: A total of 109 MIH-affected anterior teeth were treated using Icon^®^ resin infiltration (DMG, Hamburg, Germany) in this registered prospective clinical study (ClinicalTrials.gov: NCT05597956). Participants were classified as children (6–12 years) and adolescents (13–17 years) according to standard pediatric age definitions. Of these, 101 teeth were available for evaluation at the 6-month follow-up due to patient loss to follow-up. The evaluation included photographic follow-up, measurement of lesion size and color, and assessment of sensitivity. During follow-up visits, patients rated the appearance of their lesions using the FDI scale. Results: Before treatment, spectrophotometric analysis showed that lesions exhibited a reddish hue (mean a* = 2.12), were distinctly yellowish (mean b* = 23.20), and clearly differed from surrounding enamel (ΔE = 8.62). The brightness level (L* = 69.81) indicated medium-high luminosity. Lesion size was reduced by an average of 4.5 percentage points. Significant increases in L values and reductions in a* and b* components were observed, with clinically perceptible ΔE changes. Sensitivity improved in 36.6% of patients, who reported a 1–2 point decrease on the SCASS. Moreover, patients’ aesthetic perception significantly improved after Icon^®^ infiltration resin. Conclusions: Resin infiltration produced noticeable improvements in color, reduced lesion size and sensitivity, and enhanced aesthetic perception, making it a valuable treatment option for managing MIH-affected anterior teeth in children.

## 1. Introduction

Molar Incisor Hypomineralization (MIH) is a qualitative developmental defect of enamel resulting from disturbances during the mineralization or maturation stages of tooth development. It typically affects one or more permanent first molars and may also involve the permanent incisors. Diagnostic criteria were established by the European Academy of Paediatric Dentistry (EAPD) MIH in 2003 [1,2]. Clinically, MIH manifests as well-demarcated enamel opacities ranging from white to yellow or brown, often associated with enamel fragility, posteruptive breakdown, hypersensitivity, and an increased risk of caries [3]. When anterior teeth are affected, aesthetic concerns may have a profound psychosocial impact, adversely influencing children’s self-esteem and oral health–related quality of life (OHRQoL) [4].

Although MIH is highly prevalent worldwide, its etiology remains unclear and is considered multifactorial. Factors such as early childhood illnesses, perinatal complications, nutritional deficiencies, and genetic predisposition have been implicated, but no single causative agent has been confirmed [5]. Children with compromised health during their first three years of life appear to be more susceptible to severe forms of MIH. A recent systematic review confirmed the multifactorial nature of the condition, highlighting the complex interplay between environmental and biological risk factors [6].

The clinical consequences of MIH extend beyond enamel integrity. Affected children often experience pain during toothbrushing or chewing, which can compromise oral hygiene and dietary habits [7]. Enamel breakdown and hypersensitivity increase the risk of caries and result in frequent dental visits, sometimes associated with anxiety and treatment difficulties. When incisors are affected, the visible opacities frequently cause aesthetic dissatisfaction, with documented negative effects on social interactions and quality of life [8].

Management of MIH-affected teeth remains challenging because hypomineralized enamel exhibits poor mechanical properties and reduced adhesion to restorative materials. For posterior teeth, management options include fluoride varnishes, sealants, glass ionomer restorations, composite resin restorations, and preformed metal crowns. For anterior teeth, strategies such as microabrasion, external bleaching, composite resin restorations, and more recently resin infiltration have been proposed [9]. However, many of these treatments are either invasive, technique-sensitive, or provide limited and unpredictable aesthetic outcomes [10].

In addition to traditional restorative approaches, several minimally invasive alternatives have been proposed for the aesthetic management of anterior teeth affected by MIH. Microabrasion with 18% hydrochloric acid or 37% phosphoric acid, followed by the application of a remineralizing agent containing casein phosphopeptide–amorphous calcium phosphate (CPP-ACP), has shown effectiveness in improving the appearance of whitish opacities while preserving enamel structure. The etch-bleach-seal technique (acid etching, bleaching with 5% sodium hypochlorite, and sealing with a transparent resin) can be used to reduce yellow or brown stains, although its efficacy in MIH remains limited. External bleaching with hydrogen or carbamide peroxide is another non-invasive option for adolescents, but current European regulations restrict its use in children to non-effective concentrations. Composite resin restorations, with or without prior enamel removal, are suitable for masking deeper defects, though they require long-term maintenance. Recently, resin infiltration has emerged as a micro-invasive and patient-friendly technique that enhances enamel translucency and optical properties while stabilizing the surface mechanically. It has proven particularly useful for masking whitish opacities in mild to moderate MIH lesions and for improving the child’s self-esteem and oral health–related quality of life (OHRQoL) from childhood to adulthood, when more invasive treatments may be employed to achieve superior aesthetic outcomes [9,10].

Hasmun et al. (2020) [11] conducted an analysis among children aged 7 to 16 years, demonstrating that the perception of dental aesthetics was associated with changes in OHRQoL. It has been observed that some children with anterior teeth showing white, yellow, or brown spots may develop a negative self-image, disrupting their social interactions. These patients often face difficulties at school and in socializing with other children, highlighting the importance of early detection of this defect to prevent its deterioration and mitigate its impact on OHRQoL.

Defects in anterior teeth can have a significant psychosocial impact on children. A conservative approach is recommended due to the large pulp chambers, the proximity to the pulp horns, and the presence of immature gums. Moreover, a minimally invasive approach will allow for the preservation of dental structure for future restoration options. For children with poor oral hygiene, cariogenic diets, and multiple decayed teeth, aesthetic treatment of the defects should be postponed until improvements are demonstrated and the affected carious teeth are treated.

Resin infiltration was originally developed as a micro-invasive technique to arrest non-cavitated caries lesions by occluding enamel microporosities with a low-viscosity resin. Clinical trials have demonstrated that resin infiltration can significantly reduce the progression of early carious lesions compared with placebo or conventional preventive strategies [12]. Beyond caries management, it modifies the refractive index of porous enamel, reducing light scattering and masking white spot lesions. This optical effect has expanded its use to developmental enamel defects such as fluorosis and MIH [13].

Nevertheless, clinical outcomes in MIH remain heterogeneous [9]. Studies have reported partial masking of MIH opacities, with better results in white lesions than in yellow-brown ones, highlighting variability according to lesion severity and color. This inconsistency underscores the limited predictability of resin infiltration in MIH and the need for additional clinical studies with well-characterized patient populations and standardized outcome measures.

In addition to addressing these inconsistencies, this study introduces an innovative design by integrating both clinical and patient-reported outcomes to evaluate the real impact of resin infiltration on children’s perception of aesthetics and well-being. To our knowledge, it is the first clinical study to analyze the level of agreement between patient and professional aesthetic assessments using the FDI scale, providing new insights into the psychosocial relevance of this minimally invasive treatment.

The aim of this study was to evaluate the clinical effectiveness of resin infiltration in anterior teeth affected by molar incisor hypomineralization (MIH) in children and adolescents, by assessing its effects on color variation, lesion size, hypersensitivity, and patients’ aesthetic perception before and after treatment.

## 2. Materials and Methods

The study was approved by the Humans Research Ethics Committee of the Experimental Research Ethics Commission and conducted in accordance with the principles of the Declaration of Helsinki for medical research involving human subjects. The study was also registered at clinicalTrials.gov (Protocol Registration and Results System, PRS-ClinicalTrials.gov ID: NCT05597956) https://register.clinicaltrials.gov/ (accessed on 19 September 2022). Written informed consent was obtained from all participants and their parents or legal guardians. The study was conducted in accordance with the Declaration of Helsinki, and approved by the Institutional Ethics Committee of the Universite of Valencya with Verification code DHC27B313NFE71Q8 at 3 March 2022.

Study design and participants. This was a prospective interventional clinical study conducted in paediatric patients diagnosed with Molar Incisor Hypomineralization (MIH). The study was carried out at the Dental Clinic of the University of Valencia (UV) between 2022 and 2024.

The clinical trial received favorable approval from the ethics committee in March 2022, and no patient recruitment or data collection took place before this approval was granted. Although the study protocol had been fully prepared in advance for ethical review, enrollment only began once authorization was in place.

For analytical purposes, participants were categorized as children (6–12 years) and adolescents (13–17 years). Children aged 9 to 16 years were consecutively screened by calibrated examiners (M.D.C.-R. and M.A.V.-R.) according to the diagnostic criteria established by the European Academy of Paediatric Dentistry (EAPD, 2003), ensuring reliable identification of MIH lesions.

MIH severity was classified according to the criteria proposed by Chawla et al. (2008), which assess enamel opacities based on color, size, and structural integrity. Lesions were considered mild when presenting as white or creamy opacities with intact enamel, moderate when showing yellow or brown discoloration without enamel loss, and severe when post-eruptive breakdown or atypical restorations indicated structural damage.

MIH diagnosis required the involvement of at least one first permanent molar; however, participants were included in the study only if they also presented with at least one permanent anterior tooth affected by MIH, with a well-demarcated opacity clearly visible on the labial surface. Only children in good general health and with the cooperation necessary to complete clinical procedures were considered eligible. Written informed consent was obtained from all participants and legal guardians prior to study inclusion.

To ensure diagnostic consistency, both investigators (M.D.C.-R. and M.A.V.-R.) underwent a structured calibration process prior to data collection. The calibration included theoretical training on the diagnostic criteria for molar–incisor hypomineralization (MIH) and the assessment of enamel opacity color, followed by practical sessions using 30 standardized clinical photographs and 10 clinical cases not included in the study. Examiners independently evaluated these cases on two separate occasions separated by a two-week interval. Intra- and inter-examiner reliability were assessed using Cohen’s kappa statistics. A kappa value greater than 0.85 was achieved, indicating excellent agreement, according to the criteria proposed by Landis and Koch (1977) [14].

M.C.-P. supervised the entire clinical process and verified strict adherence to the study protocol throughout the trial.

Children presenting enamel defects of a different origin, such as fluorosis, hypoplasia, traumatic opacities, or amelogenesis imperfecta, were excluded to avoid diagnostic overlap. Teeth with active caries, restorations, or post-eruptive breakdown on the evaluated surface were also excluded, as were patients undergoing orthodontic treatment that could alter the anterior tooth surface. Finally, children with systemic or medical conditions that could contraindicate dental treatment or influence enamel development were not considered for inclusion.

The study was designed and reported in accordance with the TREND guidelines for a prospective single-arm interventional clinical study, to ensure transparency and methodological rigor.

Sample size of the study. The sample size for this non-randomized controlled clinical study was based on the study by Giray et al. (2018) [15] on resin infiltration in white spot lesions. Statistical parameters included an alpha risk of 0.05 and a beta risk of 0.2 for a two-tailed test. It was estimated that 100 teeth with enamel defects would be required, assuming a common standard deviation of 4.22, and accounting for an anticipated 15% loss to follow-up. The sample size calculation was performed using the publicly available GRANMO v7.11 (Institut Municipal d’Investigació Mèdica, IMIM–Hospital del Mar Research Institute, Barcelona, Spain) Online software.

Interventions. Patients who met the inclusion criteria were referred from the undergraduate Dental Clinic or the postgraduate Pediatric Dental Clinic of the UV for an initial appointment. During this appointment, parents were informed about the study and its characteristics. At this time, an evaluation of the patient was conducted to determine a global assessment of patient involvement using the Chawla index and the presence and severity of enamel defects in the incisors. Lesions of anterior teeth were clinically characterized according to the type of defect (white, yellow or brown opacity; post-eruptive breakdown), and the extent (percentage of affected labial surface). Baseline sensitivity was assessed with the Schiff Cold Air Sensitivity Scale (SCASS) [16] by applying an air stimulus for 1 s at 1 cm from the lesion.

All participants received standardized oral hygiene instructions at baseline by (M.D.C.-R.), including brushing technique reinforcement and dietary advice. Oral hygiene recommendations were reiterated at each follow-up visit. Prior to photographic documentation, teeth were gently cleaned using a prophylaxis brush and water, without polishing pastes, to remove plaque and debris while avoiding alteration of enamel surface characteristics.

Photographic records. Standardized photographic records were obtained by M.D.C.-R., with and without a polarizing filter to assess the color and surface area of the lesions. A rigid plate was used between the jaws to ensure a uniform bite and eliminate positional variations. To avoid changing the refractive index of the lesions, it was decided to keep the areas as dry as possible. A shade tab reference was included for calibration, and all photographs were taken under standardized exposure settings by the same trained operator to minimize bias.

The photographic materials were stored in JPEG format and analyzed using the GIMP 2.10 software for Mac (Berkeley, California, GNU Image Manipulation Program; The GIMP Development Team, Berkeley, CA, USA) to perform a quantitative analysis using the CIELAB system (International Commission on Illumination). The absolute values of lightness (L*), red-green color (a*), and yellow-blue color (b*) of the opacities were evaluated before and after treatment. The total color difference (ΔE) was then calculated using the following equation:

ΔE = √((ΔL)^2^ + (Δa)^2^ + (Δb)^2^)

A polarizing filter (Polar_Eyes^®^, Emulation, Los Angeles, CA, USA) and a Yukiko Ring Softbox Speedlight Round style flash diffuser (Monllack, Shenzhen, China) were utilized. The photographs were taken with a Canon 600D camera (Canon Inc., Tokyo, Japan), using a 60 mm macro lens (Canon EF-S 60 mm f/2.8 Macro USM; Canon Inc., Tokyo, Japan), a Meike MK-14EXT ring flash (Meike Global, Shenzhen, China), and a circular diffuser. The camera settings were as follows: aperture F22, shutter speed 1/125, and ISO 100.

Lesion size was determined using the same standardized photographs. The examiner delineated the opacity at baseline (T0) and after treatment (T6) using the free selection tool in GIMP. Lesion size was expressed as a percentage of the total labial surface to allow standardized comparisons. All measurements were carried out by a single calibrated examiner to reduce measurement bias.

**Figure 1:**
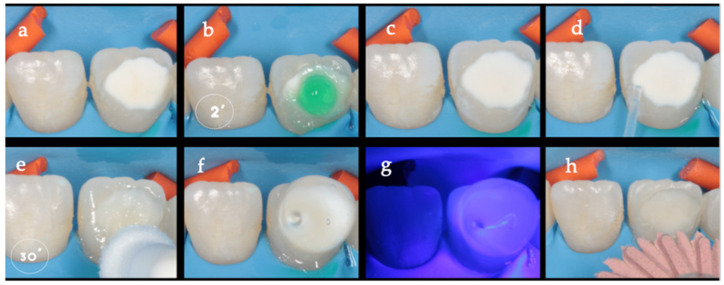
Modified resin infiltration protocol for anterior MIH opacities. The clinical sequence (from left to right) (**a**) illustrates the resin infiltration procedure performed under: (**a**) rubber dam isolation, shown from right to left and from top to bottom. The sequence begins with the baseline appearance of the MIH-affected anterior tooth. (**b**) A 15% hydrochloric acid gel (Icon-Etch) is then applied to the hypomineralized enamel for 2 min to remove the superficial hypermineralized layer, followed by thorough rinsing and (**c**) air drying. (**d**) Absolute ethanol (Icon-Dry) is subsequently applied for 30 s to desiccate the lesion and facilitate resin penetration. (**e**) A low-viscosity infiltrant resin (Icon-Infiltrant) is then applied and maintained on the enamel surface for a prolonged period of 30 min as part of the modified protocol to enhance penetration into hypomineralized enamel. Excess resin is removed, and the material is light-cured. (**f**) Glycerin gel is applied to eliminate the oxygen-inhibited layer, followed by an additional (**g**) light polymerization. The sequence concludes with (**h**) finishing and polishing of the treated surface, demonstrating improved optical integration of the lesion with the surrounding enamel.

Resin infiltration process. Resin infiltration, performed in accordance with the manufacturer’s instructions (Icon^®^, DMG, Hamburg, Germany), was carried out on the teeth using the following procedure: firstly, the 15% hydrochloric acid gel, known as Icon-Etch, was applied to the enamel lesions three times for a total duration of 120 s. This was then followed by rinsing with an air-water spray for 30 s to remove the acid. The water present within the microporosities of the lesions was then chemically removed using absolute ethanol (Icon-dry) for a duration of 30 s, followed by air drying. Subsequently, the infiltrant resin (Icon-infiltrant) was applied to the surface for a period of 30 min (Figure 1). The prolonged application was designed to promote resin penetration in all types of opacities. Excess resin was removed using dental floss, then polymerized for 40 s with the VALO™ Cordless Kit (Ultradent Products Inc., South Jordan, UT, USA). Glycerin (Ultradent Products Inc., South Jordan, UT, USA) was applied to eliminate oxygen-inhibited layers, followed by an additional polymerization and polishing with Eve™ Diacomp Plus discs (EVE Ernst Vetter GmbH, Keltern, Germany) to remove any remaining excess. The infiltration procedure was performed by M.D.C.-R., under rubber dam isolation. M.A.V.-R. assisted in case preparation, patient management, and recording of treatment times. All clinical interventions were supervised by M.C.-P., who ensured the uniform application of the protocol and verified that no deviations occurred between cases.

Objective outcomes included spectrophotometric and digital image-based CIELAB color measurements and quantitative lesion size assessment. Subjective outcomes comprised dental hypersensitivity evaluated using the SCASS and aesthetic perception assessed with the FDI scale. SCASS scores were recorded based on patient responses to a standardized air stimulus, while FDI scores were assigned independently by calibrated examiners and by patients using predefined categorical criteria. All clinical, photographic, colorimetric, sensitivity, and aesthetic assessments were performed by the same calibrated examiner to ensure intra-examiner reliability (MD.C-R). Examiner calibration achieved Cohen’s kappa values > 0.85 for categorical assessments and intraclass correlation coefficients > 0.90 for continuous measurements.

Colour determination using a spectrophotometer. The spectrophotometer (Easyshade V, Vita Zahnfabrik, Bad Säckingen, Germany) was calibrated before each measurement. The probe tip was positioned in the center of the opacity. To ensure consistent positioning, custom silicone positioning devices were fabricated for each patient, allowing reproducible placement of the probe across visits. Spectrophotometric measurements were taken by M.D.C.-R., using custom silicone guides fabricated for each patient to ensure precise probe positioning. The methodology and calibration procedures were reviewed by M.C.-P. prior to data acquisition to guarantee consistency. Intra- and inter-examiner reliability tests showed excellent agreement, with intraclass correlation coefficients (ICC) exceeding 0.90, confirming the reproducibility of the measurements across sessions.

Determination of sensitivity prior and post treatment. Dental hypersensitivity was assessed using the SCASS. Sensitivity measurements were performed under standardized clinical conditions at baseline (T0), immediately after resin infiltration, and at each follow-up visit. All SCASS assessments were conducted before rubber dam placement, with the tooth fully exposed and isolated using cotton rolls to avoid contamination from saliva. The air stimulus was applied for 1 s at a standardized distance of 1 cm from the labial surface using a dental air syringe, while adjacent teeth were shielded with cotton rolls to prevent stimulus dispersion. Sensitivity assessments were always performed by the same calibrated examiner to ensure reproducibility. To ensure that sensitivity originated exclusively from the treated tooth, adjacent teeth were physically isolated during testing and individually assessed when necessary, confirming the absence of hypersensitivity responses in neighboring teeth.

Impact on esthetic perception. The aesthetic appearance of the teeth before and after treatment was evaluated using the color-matching criteria of the Fédération Dentaire Internationale (FDI) [17]. The FDI aesthetic scale was independently applied by two calibrated dental professionals (M.D.C.-R. and M.A.V.-R.), both experienced in pediatric restorative dentistry. Examiner calibration was performed prior to the study, achieving a Cohen’s kappa value > 0.85 for aesthetic assessment. In parallel, patients were asked to rate their own aesthetic perception using the same FDI criteria after standardized photographic documentation at each evaluation time point. Patients’ aesthetic perception was evaluated using the aesthetic criteria of the Fédération Dentaire Internationale (FDI). Participants were asked to rate the aesthetic appearance of the resin-infiltrated teeth using a five-point quality scale as follows: (1) clinically excellent/very good, indicating a good color match with no perceptible difference in tone or translucency; (2) clinically good, indicating minor deviations; (3) clinically adequate/satisfactory, indicating a clear but acceptable deviation that does not affect aesthetics; (4) clinically unsatisfactory; and (5) clinically poor, considered unacceptable and requiring replacement.

To evaluate the teeth as accurately as possible at each visit, follow-up photographs were taken. The procedure was always the same; patients were positioned in the specially designed photographic setup for this study, seated on a stool with their heads aligned in the natural plane, at a distance of 30 cm from the camera’s lens. To prevent the alteration of the refractive index of the lesions, the same measurements were used for all photographs, and the parameters set manually on the camera remained consistent throughout the process. The follow-up was conducted prior to treatment, immediately afterward, at 15 days, at 3 months, and at 6 months. The assessment of patients’ aesthetic perception using the FDI criteria was jointly conducted by M.D.C.-R. and M.A.V.-R.

Statistical analysis. The original protocol specified three categories: white, yellow, and brown. During the initial statistical analysis, however, we observed that the yellow and brown opacities did not differ significantly from each other in any of the evaluated parameters and showed very similar clinical and behavioral characteristics. In contrast, white opacities demonstrated a clearly distinct pattern. Given the absence of meaningful differences between yellow and brown lesions, and in order to increase statistical power and avoid redundant comparisons, the two groups were combined for analysis.

Inferential analysis encompassed several approaches. Linear regression models with generalized estimating equations (GEE) were used to analyze changes in the CIELAB color parameters (L*, a*, b*). Multiple comparisons were adjusted using the Bonferroni criterion. For the analysis of ΔE, the same models were applied. The Wald chi-squared test was used to examine baseline lesion characteristics. Changes in sensitivity were assessed using the Friedman and Wilcoxon tests. Results are presented with 95% confidence intervals. Dropouts and missing data were recorded, and sensitivity analyses were performed to evaluate their potential impact. Adjusted models were used to explore possible confounders, including age, sex, and baseline lesion characteristics. A significance level of *p* < 0.05 was considered statistically significant.

To assess the FDI index, the Friedman test and Wilcoxon test were applied, as these analyses were conducted at the patient level and the observations were repeated measures at the patient level. Finally, agreement between the FDI index reported by the patient and the dentist was evaluated using the linearly weighted Kappa statistic, along with its 95% confidence interval. Interpretation of the kappa values followed the Landis-Koch criteria.

## 3. Results

**Figure 2:**
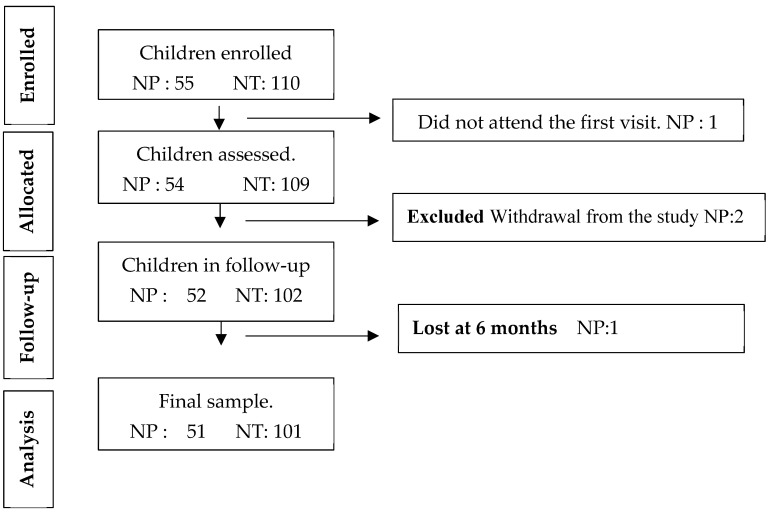
TREND flow diagram. Abbreviations used in this figure. NP: number of patients; NT: number of teeth.

During the study period, four participants (7.3%) were lost to follow-up due to missed appointments or voluntary withdrawal, resulting in 101 teeth completing the 6-month evaluation. A total of 55 patients with 110 MIH-affected anterior teeth were initially recruited. All underwent the same infiltration protocol and follow-up. During the study, four participants (7.3%) were lost to follow-up: two missed their scheduled visits and two withdrew voluntarily (Figure 2). Consequently, 51 patients and 101 teeth completed the 6-month evaluation.

**Figure 3:**
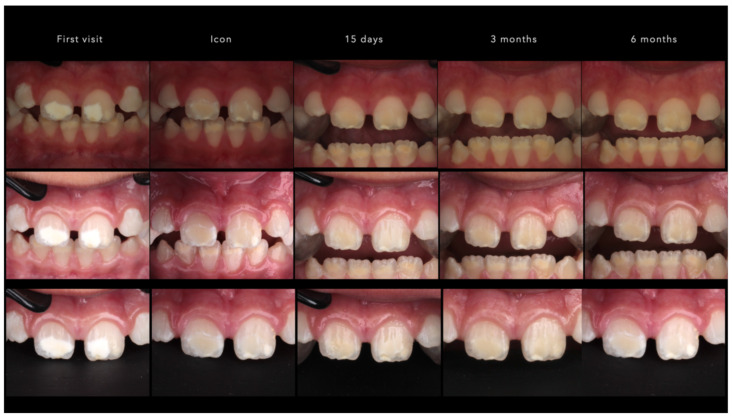
Standardized follow-up photographs at T0 (before treatment), T1 (immediately after treatment), T2 (15-day follow-up), T3 (3-month follow-up), and T6 (6-month follow-up).

As shown in Table 1, participants ranged in age from 9 to 16 years (mean = 12.6 ± 2.4 years). Thirty-seven percent (n = 20) were male and 63% (n = 34) female. The final analysis included 109 teeth: 78 (73.4%) central incisors, 24 (22.9%) lateral incisors, and 7 (3.7%) canines. Representative standardized clinical images illustrating these baseline characteristics are shown in Figure 3.

The mean Chawla index score among patients was 3.5 ± 1.2. The mean number of affected teeth per patient was 2.6 ± 1.3 incisors and 3.0 ± 1.2 molars. According to the opacity color, 64 lesions were classified as white (51 CI, 13 LI), 37 as yellow (25 CI, 11 LI, 1 C), and 8 as brown (2 CI, 6 C).

### 3.1. Lesion Areas

At baseline (T0), defects covered 17.9% of the total labial Surface. At 6 months (T6), this decreased to 13.4%, corresponding to a mean reduction of 4.5 percentage points (95% CI −5.8 to −3.2; *p* < 0.001), indicating a clinically relevant improvement.

### 3.2. Color Parameters

At baseline, spectrophotometric measurements revealed hypomineralized enamel with slightly reddish (a* = 2.12) and markedly yellowish (b* = 23.20) tones, and a mean lightness (L* = 69.81). The mean color difference from sound enamel was ΔE = 11.13. Digital image analysis produced comparable results (L* = 65.52, a* = 5.65, b* = 13.91; ΔE = 13.47). To avoid redundancy, spectrophotometric data are presented as the main outcome, with digital analysis included for confirmation (Table 2).

**Figure 4:**
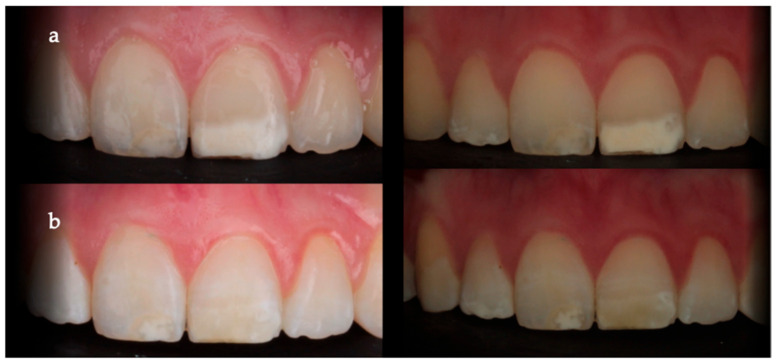
Representative visual example of an anterior tooth affected by MIH: (**a**) baseline appearance before resin infiltration and (**b**) appearance after resin infiltration at 6-month follow-up, showing improved color integration with surrounding enamel.

After infiltration, both methods demonstrated a significant reduction in ΔE (*p* < 0.001), confirming a perceptible color improvement, an example of this is shown in Figure 4.

### 3.3. Subgroup Analysis by Lesion Color

When stratified by lesion color (white vs. yellow/brown), baseline L* values were significantly higher in white lesions, while a* and b* values were lower (Table 3). After infiltration, white opacities showed a decrease in L, whereas yellow-brown opacities exhibited a significant reduction in a* and b* values.

### 3.4. Sensitivity

**Figure 5:**
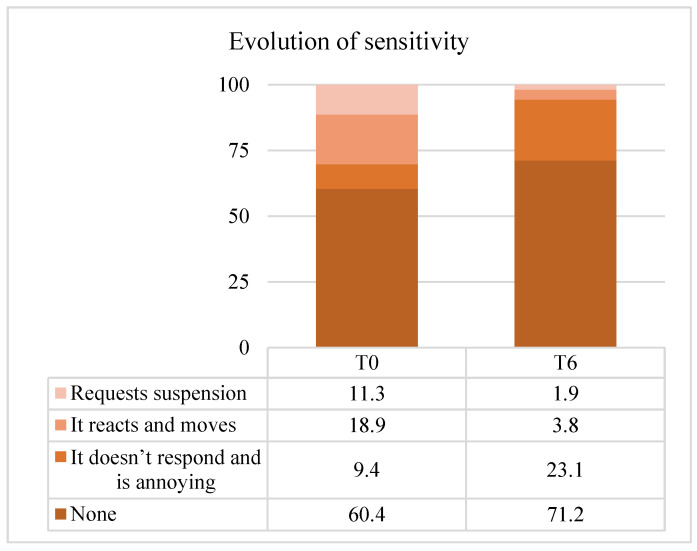
Changes in dental sensitivity (SCASS scores) after infiltration.

Sensitivity outcomes showed significant improvement. According to the Wilcoxon test, SCASS scores were significantly lower after infiltration (*p* < 0.001). Initially, most patients reported moderate sensitivity; at T6, most reported none or minimal sensitivity. Specifically, 61.5% of patients showed no change, 28.8% improved by 1 point, 7.7% by 2 points, and only one patient worsened by 1 point (Figure 5). The median change was −1 (IQR 0 to −1), corresponding to a moderate effect size (r = 0.34).

### 3.5. Esthetic Perception (FDI Index)

Children’s aesthetic self-perception significantly improved after treatment (*p* < 0.001, Friedman test), shifting from predominantly unsatisfactory ratings at baseline to good or excellent at follow-up. At T0, patients tended to rate their appearance more critically than the professional, but by T6 this tendency reversed, with the professional being slightly more critical.

### 3.6. FDI Assessment Agreement Between Patient and Professional

The agreement between patient and professional FDI classifications was generally weak (Table 4), with variable concordance across evaluation times: patients were initially more negative (T0–T1), balanced at T2, and progressively more positive at T3–T6.

Patients’ self-assessed aesthetic perception also showed a statistically significant improvement after resin infiltration. The mean aesthetic score (±SD) increased from 2.41 ± 0.63 at baseline to 4.17 ± 0.56 at 1 month and 4.24 ± 0.48 at 6 months (Friedman test, *p* < 0.001). Pairwise comparisons using the Wilcoxon test revealed significant differences between baseline and both follow-up periods (*p* < 0.001), whereas no significant variation was observed between the 1- and 6-month evaluations (*p* = 0.417). These findings indicate that most of the improvement in perceived aesthetics occurred immediately after treatment and remained stable throughout the observation period.

## 4. Discussion

Significant post-treatment changes were observed in the CIELAB parameters, with ΔE values exceeding the clinical perceptibility threshold of 3.7 units. This confirms a noticeable aesthetic improvement, consistent with previous reports [18,19,20]. Baseline differences between white and yellow/brown opacities were confirmed: white lesions showed higher L* values (brightness) and lower a* and b* values compared with darker opacities. After infiltration, yellow–brown opacities exhibited greater reductions in reddish and yellowish tones, in agreement with Khanna et al. (2020) [21], suggesting that optical responses vary depending on baseline chromatic characteristics.

The objective CIELAB colorimetric findings provide a quantitative framework for interpreting the clinical and perceptual outcomes observed in this study. In particular, increases in L values reflect enhanced enamel brightness, while reductions in a* and b* values indicate attenuation of reddish and yellowish chromatic components commonly associated with MIH opacities. These changes correspond with the clinically perceptible ΔE values observed and support the subjective improvements reported by patients and clinicians. The integration of instrumental colorimetric analysis with lesion size reduction and hypersensitivity outcomes allows for a more comprehensive interpretation of treatment efficacy.

At baseline, over one-third of participants exhibited extensive lesions covering more than two-thirds of the tooth Surface. After 6 months, a significant mean reduction of 4.5% in lesion area was recorded (95% CI: −5.8 to −3.2). Although smaller than the 50% reduction reported by Warner et al. (2021) [22] in white opacities, this difference may reflect the higher proportion of complex yellow/brown lesions in our sample. Similar trends were observed by Gu et al. (2021) [23] and Altan et al. (2023) [24], confirming that infiltration can effectively reduce lesion size, though its success depends on initial color and extension.

Hypersensitivity outcomes also improved significantly, with 36% of patients reporting decreased discomfort and an overall moderate effect size (Wilcoxon r = 0.34). These results are consistent with Diago et al. (2021) [25] and Brescia et al. (2022) [26], who demonstrated that resin infiltration provides a sustained reduction in sensitivity compared with other conservative approaches such as fluoride varnish or CPP-ACP. This improvement is clinically relevant and contributes to better oral hygiene, eating comfort, and social confidence.

Because MIH in anterior teeth primarily generates aesthetic rather than functional concerns, assessing patients’ self-perception was essential. The FDI aesthetic scale revealed a marked shift from initial dissatisfaction to ratings between “good” and “excellent” at follow-up, consistent with Mazur et al. (2018, 2022) [27,28]. Brescia et al. (2022) [26] observed less favorable long-term outcomes, underscoring the influence of lesion depth and location. Interestingly, while professionals initially rated appearance more positively than patients, this trend reversed at six months. This longitudinal divergence suggests that patients increasingly valued the aesthetic and emotional benefits of treatment. To our knowledge, this is the first study to evaluate both patient and professional satisfaction over time, emphasizing the relevance of patient-reported outcomes as a complement to clinical indices and the importance of patient-centered care.

These results reinforce the need to integrate patients’ expectations into clinical decision-making. As highlighted by Warner et al. (2021) and Rodd et al. (2021) [22,29], clinicians and patients often differ in their perception of success: while professionals focus on the objective reduction of opacities, patients frequently value subtle but visible improvements that restore confidence in their smile. Incorporating patient-reported measures such as OHRQoL questionnaires could therefore provide a more comprehensive understanding of treatment impact in young populations.

The combined improvement in color, lesion size, sensitivity, and aesthetic perception supports the clinical and psychosocial relevance of resin infiltration as a conservative treatment for anterior MIH lesions. However, the variability of outcomes, particularly in darker or extensive defects, suggests that infiltration should be viewed as part of a broader therapeutic spectrum, potentially complemented by restorative or bleaching techniques.

This study is of particular importance as it represents one of the few clinical investigations focusing exclusively on anterior teeth affected by MIH in children and adolescents. In contrast to previous research that primarily addressed posterior teeth or short-term color outcomes, such as the studies by Warner et al. (2022) [22], Gu et al. (2021) [23], and Altan et al. (2023) [24], the present work provides a comprehensive assessment that integrates objective parameters (color change, reduction in lesion size, and improvement in sensitivity) with subjective measures (patient-reported aesthetic perception). Interestingly, the findings revealed that patient and professional perceptions do not always align, underscoring the importance of integrating the patient’s perspective when assessing treatment success. These aspects highlight the clinical relevance and originality of the study, reinforcing the importance of minimally invasive and patient-centered approaches for the management of MIH lesions in visible anterior teeth.

This study presents some limitations. The absence of a control group precludes direct comparison with the natural progression of MIH lesions or with alternative treatments. The 6-month follow-up may not reflect the long-term stability, and extended infiltration times likely improved resin penetration in deeper lesions but may have provided limited additional benefit for shallower ones, thereby increasing variability. Despite the use of objective spectrophotometry, visual assessment remains partly subjective.

Future studies should include control groups, longer follow-ups, and standardized outcome measures. The incorporation of patient-reported outcomes (satisfaction, self-esteem, OHRQoL) and direct comparisons with other minimally invasive techniques (microabrasion, bleaching, or combined protocols) will help refine clinical guidelines and improve individualized management of MIH lesions.

## 5. Conclusions

Within the limitations of this prospective clinical study, resin infiltration was associated with improvements in color masking, lesion size reduction, dental hypersensitivity, and patient-reported aesthetic satisfaction in anterior teeth affected by molar–incisor hypomineralization. While patients reported increased satisfaction with dental appearance, no direct psychosocial or psychological outcomes were assessed. Therefore, conclusions regarding psychosocial well-being should be interpreted with caution. Further controlled studies with longer follow-up periods and validated patient-reported outcome measures are required to confirm the clinical effectiveness and broader impact of this minimally invasive approach.

## Figures and Tables

**Table 1:** Baseline (T0) characteristics of the study sample.

Category	Variable	Values
Demographics	Mean Age (years)	12.6 ± 2.4
	Gender	Male: 37%; Female: 63%
Teeth	Distribution	CI: 73.4%; LI: 22.9%; C: 3.7%
	Upper/lower jaw	81.7%/18.3%
Lesion characteristics	Color	White: 64 (58.7%); Yellow: 37 (33.9%); Brown: 8 (7.4%)
	Chawla score	3.5 ± 1.2
	Size (% of vestibular surface)	17.9%
Colorimetry (T0)	Spectrophotometer L*	69.81
	Spectrophotometer a*	2.12
	Spectrophotometer b*	23.20
	ΔE Spectrophotometer	11.13
	Digital image L*	65.52
	Digital image a*	5.65
	Digital image b*	13.91
	ΔE Digital image	13.47

CI: central incisor; LI: lateral incisor; C: canine.

**Table 2:** Changes in CIELAB parameters (L*, a*, b*, ΔE) after resin infiltration.

Method	Parameter	T0 Mean (SD)	T6 Mean (SD)	Mean Change (Δ)	95% CI Δ	Min–Max (T0)	Min–Max (T6)	*p*-Value
Spectrophotometer	L*	69.81 (±12.4)	76.54 (±10.8)	+6.73	5.2–8.3	31.0–85.3	54.3–89.7	<0.001
	a*	2.12 (±2.6)	1.49 (±2.7)	−0.63	−1.2–−0.1	0.0–11.4	0.0–12.0	0.042
	b*	23.20 (±8.2)	23.05 (±7.9)	−0.15	−0.8–0.6	7.8–42.1	5.5–48.0	0.68
	ΔE	11.13 (±5.8)	11.46 (±6.1)	+0.33	−0.6–1.2	0.3–37.6	0.6–38.2	0.47
Digital image	L*	65.52 (±13.5)	60.14 (±12.6)	−5.38	−6.7–−4.0	21.8–83.8	27.7–80.5	<0.001
	a*	5.65 (±3.1)	5.41 (±2.9)	−0.24	−0.7–0.2	0.4–15.6	1.0–17.4	0.29
	b*	13.91 (±7.4)	11.26 (±6.8)	−2.65	−3.4–−1.9	0.1–32.5	0.5–35.0	<0.001
	ΔE	13.47 (±6.2)	12.12 (±5.9)	−1.35	−2.1–−0.6	0.6–35.3	0.7–34.1	0.002

n: number of teeth. ΔE ≥ 3.7 indicates clinically perceptible change.

**Table 3:** Changes in CIELAB parameters (L*, a*, b*, ΔE) according to lesion color (white vs. yellow/brown opacities).

Group	Method	Parameter	T0 Mean (SD)	T6 Mean (SD)	Mean Change (Δ)	95% CI Δ	Min–Max (T0)	Min–Max (T6)	*p*-Value
White	Spectrophotometer	L*	71.67 (±9.2)	76.90 (±8.7)	+5.23	3.8–6.7	48.7–85.3	54.3–89.7	<0.001
		a*	1.21 (±1.4)	1.36 (±1.6)	+0.15	−0.2–0.5	0.0–5.1	0.0–7.4	0.41
		b*	19.92 (±6.8)	20.45 (±7.1)	+0.53	−0.5–1.5	7.8–42.1	1.6–57.9	0.31
		ΔE	9.90 (±4.7)	10.65 (±5.1)	+0.75	−0.3–1.8	0.3–29.9	0.6–38.2	0.16
	Digital image	L*	67.83 (±10.5)	61.37 (±9.9)	−6.46	−7.8–−5.1	38.1–83.8	37.7–79.2	<0.001
		a*	4.66 (±2.7)	4.00 (±2.5)	−0.66	−1.2–−0.1	0.4–15.6	1.0–14.2	0.028
		b*	10.84 (±5.9)	7.69 (±5.1)	−3.15	−4.1–−2.2	0.1–32.5	0.5–23.4	<0.001
		ΔE	12.17 (±5.5)	10.65 (±4.8)	−1.52	−2.6–−0.5	0.6–30.3	0.6–29.8	0.004
Yellow/Brown	Spectrophotometer	L*	67.23 (±11.1)	76.07 (±9.6)	+8.84	6.9–10.8	31.0–82.3	57.5–86.2	<0.001
		a*	3.38 (±2.8)	1.94 (±2.6)	−1.44	−2.0–−0.9	0.0–11.4	0.0–11.5	<0.001
		b*	27.73 (±8.5)	27.40 (±8.2)	−0.33	−1.1–0.5	9.9–42.1	11.3–45.3	0.42
		ΔE	12.83 (±6.1)	12.52 (±6.0)	−0.31	−1.2–0.6	0.3–37.6	1.7–34.8	0.52
	Digital image	L*	61.78 (±12.3)	58.51 (±11.8)	−3.27	−4.8–−1.7	21.8–78.1	27.7–80.5	<0.001
		a*	7.13 (±3.2)	7.27 (±3.3)	+0.14	−0.4–0.6	1.5–13.5	2.3–17.4	0.61
		b*	18.08 (±6.9)	15.97 (±6.2)	−2.11	−3.1–−1.2	2.5–29.6	3.4–35.0	<0.001
		ΔE	15.18 (±6.3)	13.62 (±6.0)	−1.56	−2.6–−0.5	1.2–35.3	1.7–34.6	0.005

**Table 4:** Concordance between patient and professional FDI appearance ratings: percentage of agreement, Kappa index, 95% confidence interval, and qualitative interpretation.

	%	Kappa	IC95%	Interpretation
T0	46.1	0.26	0.12–0.41	Weak
T1	42.3	0.17	0.00–0.38	Poor
T2	45.1	0.24	0.00–0.48	Weak
T3	45.1	0.31	0.12–0.50	Weak
T6	41.2	0.15	0.03–0.28	Poor

## Data Availability

The data will be made available upon reasonable request to the authors.

## Abbreviations

The following abbreviations are used in this manuscript:

EAPD	European Academy of Paediatric Dentistry
MIH	Molar incisor hypomineralization
FDI	Fédération Dentaire Internationale
SCASS	Schiff Cold Air Sensitivity Scale
OHRQoL	Oral Health-Related Quality of Life
UV	University of Valencia
GEE	Generalized estimating equations
CI	Central incisor
LI	Lateral incisor
C	Canine
